# Medical Opinion and Movement

**Published:** 1910-10-15

**Authors:** 


### MEDICAL OPINION AND MOVEMENT.

### Drainage in Gynæcological Operations.

IN view of the tendency of gynæcologists to renounce abdominal drainage in laparotomies or to replace it in some cases by vaginal drainage, Drs. Hartmann and Metzger have made a detailed report upon 997 laparotomies carried out by them, showing the varying results obtained by employing abdominal or vaginal drainage or no drainage at all. 268 laparotomies were for uterine fibromata, with a total mortality of 4.1 per cent. Of these seventy-five cases were drained, with a mortality of 2.66 per cent., and 195 cases were not drained, with a mortality of 4.6 per cent. The drainage was chiefly abdominal, but in nineteen cases vaginal, without any death. Cases of operation on the appendages amounted to 446. Of these fifty-one were not drained, with a mortality of 3.9 per cent., and 174 cases drained gave a mortality of 3.44 per cent. Seventy-seven cases were drained by the abdomen, with 2.59 per cent. mortality, seventy-eight by the vagina, with 3 per cent., and nineteen by both ways with 5.26 per cent. mortality. The remaining operations for extra-uterine pregnancy and other conditions showed the same result in favour of drainage. The authors are moreover in favour of abdominal as against vaginal drainage. There is less tendency to febrile reaction with the former, and the abdominal drain can be retained as long as may be necessary, whereas the vaginal drain falls out within the first ten to fifteen days. Moreover they do not consider that the drainage tends to increase the liability to abdominal hernia. In cases in which this has occurred the drain was not the site of the hernia, but always the umbilical region.

### Surgical Treatment of Exophthalmic Goitre.

AT the recent French Surgical Congress held at Paris one of the chief subjects chosen for discussion was the surgical treatment of exophthalmic goître. The discussion was initiated by Dr. Delore, of Lyons. He discussed the various operative means employed in an impartial manner, and considers the method of choice is sub-capsular semi-thyroidectomy on the posterior surface of the lobe. He favours this method chiefly because it is the most sure means of avoiding injury to the parathyroid glands. He admits that whereas the condition is generally ameliorated by operation, a complete cure is rare. Sympathectomy also is followed by diminution of the exophthalmia and improvement in the psychic condition, and is especially indicated in conditions in which the thyroid is not greatly enlarged, but there is marked exophthalmia. Dr. Lenormant is more optimistic and holds that surgical intervention can effect a cure in at least 75 per cent. of cases if it is carried out at an early stage of the disease. He deplores the fact that physicians do not apply for surgical aid till the cases are already in a seriously advanced condition. Many others spoke in the same strain, and while it was admitted that operation is not justifiable when the symptoms of the disease are as yet slight, it was urged that the disease should not be allowed to assume a serious aspect before the surgeon is consulted. Sir Victor Horsley took part in the discussion. He holds that operation is indicated in cases of moderate severity treated medically for six weeks without improvement.

### Ultimate Prognosis of Exophthalmic Goitre.

AN exhaustive paper of more than usual interest is that of Dr. Hale White in the current number of the *Quarterly Journal of Medicine*. By following up the history of a large number of exophthalmic patients seen in hospital and in private practice he endeavours to ascertain what is the ultimate end of this disease, and whether expectant treatment is preferable to the operative measures now so freely advocated by many surgeons. Dr. Hale White is so well known as a thoroughly sound clinician with no prejudice against the claims of modern surgery that he is well fitted for the position of arbitrator between the extremists of either side. Of 169 cases admitted during twenty years to Guy's Hospital 21 died in the hospital and 54 of the remainder have been traced; the remaining 75 cannot be accounted for. Of the 54, five were operated upon, leaving 49 who received expectant treatment and left the hospital. Eight have died out of these 49, whereas an actuarial estimate based on their ages and the length of time elapsed since their discharge shows that the expected mortality would be five. Amongst 53 private patients traced there have been seven deaths, as against three by actuarial computation. So that roughly speaking the mortality seems to have been just about twice that of an equal number of healthy women of the same age distribution. On the other hand, this conclusion must be accepted with qualification, for there were but few slight cases included in the list; amongst such the mortality would naturally be less.

WITH regard to 40 hospital patients now alive and never operated upon, 26 have done very well, 12 are fairly well, and two only are not well, though even these say they are better than formerly. Of 47 private patients in the same classification, 35 are very well, nine are fairly well, and three have not done well, but of these one refused all treatment and another relapsed after discontinuing her treatment. It is also noteworthy that some of the most serious and severe cases have ultimately done among the best. Then of eight hospital cases operated upon three died as an immediate result; of the five others three are better but not well, and two are perfectly well. Similarly of the private patients, one died on the operating table, and neither of the other two is cured. Dr. Hale White admits that Kocher, Mayo, and others show better results of operative treatment than this, but he believes that they select the early and mild cases such as are most likely to do well in any case. Many of the patients in the series now analysed were so ill as to forbid operation entirely, yet many recovered. He thinks it possible that operation may hasten cure; if so, the justification is greater amongst wage-earners than others. He declares that those who decline operation cannot be considered unreasonable. The author will not commit himself to any deductions about treatment, except that rest is the most important item and feeding the next.

### Oxygen and Carbon Dioxide Inhalations.

On the supposition that carbonic acid gas plays an important part in the regulation of the respiratory function and influences also the cardiac activity and tonicity of the vascular system, Dr. E. Levy, of Florence, has conceived the idea of employing mixtures of oxygen and carbon dioxide in cases in which a prompt and energetic stimulation of the respiratory nerve centres is required—cases of chloroform intoxication, surgical shock, etc. Inhalations of such mixtures have already been used to combat the ill-effects produced by high altitudes and low barometric pressures in the course of an ascension up a mountain or in a balloon. Dr. Levy first carried out a series of experiments on animals. By means of nitrites, chloroform, or morphia they were brought into a condition of severe bulbar depression with slowing or complete suspension of the respiratory function. They were then made to inhale a mixture of oxygen and carbon dioxide (the latter in the proportion of 10 to 30 per cent.). This was rapidly followed by a return of respiration. In view of these facts the author has applied the treatment in a number of comatose or semi-comatose conditions from various causes, using a mixture of oxygen and 5 to 20 per cent. carbon dioxide. The treatment is always followed by a rapid increase in the frequency and amplitude of the respiratory movements, accompanied by increased cardiac activity and vascular tone.

It has been suggested by Henderson that surgical shock depends to a great extent upon insufficiency of carbonic acid in the blood in consequence of excessive elimination of this gas through exposed visceral surfaces and by the lungs during the "hyperpnoïc phase" of the narcosis. In such cases Dr. Levy advises 30 per cent. carbon dioxide in order to insure a prompt and energetic effect. The inhalation of a large proportion of oxygen (70 per cent.) at the same time excludes any danger from this proceeding. These means should be resorted to as soon as the respiratory movements begin to show signs of irregularity. In pursuance of this theory of insufficiency of carbonic dioxide the author suggests that the rapid return to consciousness observed in anæsthesia with the circulation artificially reduced is due to the sudden return to the general circulation of a large quantity of blood rich in carbonic acid, and he thinks that the inhalation of a mixture of oxygen and 10 to 15 per cent. carbonic acid at the end of an operation would produce a more rapid return of the patient to consciousness, and would also diminish the tendency to post-anæsthetic vomitting.

### Hæmophilia Neonatorum.

That very mysterious bleeding of the newly born which is occasionally seen, and is generally attributed, for want of any more accurate information, to hæmophilia, has been hitherto but little amenable to treatment. In the *American Journal of Medical Sciences* Welch reports eight such cases treated with human blood serum, with only one death. He was led to try this by observing that after actual transfusion with blood the transfused red corpuscles are largely destroyed, some in the vessels and some in the bone marrow, where leucocytes can be seen engulfing them. The quantity varied between 10 and 65 c.c. per diem, about 6 to 10 c.c. being given at a time. It was observed that the temperature in each case came rapidly down to normal, and the loss of weight was also checked at once; indeed, in three case an immediate gain in weight was noticed. In the one fatal case death took place seven weeks after all bleeding had ceased, and is ascribed to other causes. The author believes that physiologically serum is a splendid food when thus injected straight into a vein: no after-effects are observed when human serum is thus employed. He suggests that it may prove of the greatest value in the exhaustion of infantile diarrhœa, and in other acute infective conditions in which disturbances of digestion co-operate in reducing a child's strength so greatly as to contribute largely to a fatal issue.

### The Pulse and Epileptic Seizures.

In his Goulstonian lectures Dr. A. E. Russell has supported the view that the existing cause of the epileptic fit is a sudden lack of arterial blood in the brain, and from certain observations of his own he puts forward the proposition that in some cases at least there is a cessation of the pulse before the fit. Drs. Gibson, Good, and Penny have succeeded in obtaining from five epileptic subjects pulse tracings which show the transition from the normal to the epileptic stage and are published in the *Quarterly Journal of Medicine*. No cessation of the pulse is visible on any of the tracings, though in one case the pulse at the wrist as palpated by the finger seemed to have disappeared; but the authors point out that in the spasms of epilepsy the anterior tendons of the wrist may become so tautened as to conceal the pulse-wave to the examining finger. They confirm the conclusion reached by Munson that there is a slight quickening of the pulse immediately preceding the fit, but the graphic records taken from the brachial artery show that there is no alteration of the pulse sufficient to affect the amplitude of the wave up to the point when clonic convulsions prevent its being properly recorded; in more than one record the fit was definitely in progress before the record was thus interfered with. They find no such lowering of general blood pressure as to suggest that cerebral anæmia from any general cause can produce a convulsion. They do not wish, however, to deny that stoppage of the pulse is a possible cause of epilepsy; but they look for the mechanism of the fit rather to a local cause in the brain, such as a vasomotor spasm, than to a general cause such as lowering of blood pressure from cardiac inhibition or splanchnic dilatation.

### Suprarenal Hæmorrhage in Infants.

Litzenberg and White publish in the *Journal of the American Medical Association* an analysis of 119 cases of hæmorrhage of the suprarenal bodies in infants, from which they conclude that hæmorrhage in these organs is more common than in others. The primary cause of this is due to the close proximity of the suprarenals to the vena cava, which makes congestion easy. Moreover, their anatomical structure favours bleeding. This usually occurs in the internal cortical zone, because of its vascularity and the anastomotic arrangement of the vessels. The bleeding always follows congestion, whether active or passive. The latter may be caused by anything which favours venous stasis, such as difficult labour, thrombosis, etc. Active congestion results from infection or toxæmia. In two cases reported Friedlander's pneumo-bacillus was noticed, and in five others bacteria of different kinds were found. Thus infection is a cause of suprarenal hæmorrhage. Death may result either from loss of blood or from changes in the physiological activity of the organ.

### Spirochæte of Syphilis in Umbilical Cord.

Some observations carried out by Emmons on the diagnostic value of the search for Spirochæta pallida in the umbilical cord of the newborn, and reported in the *Boston Medical and Surgical Journal*, show that a negative result does not by itself exclude the existence of syphilis in the child. Only on rare occasions has he been able to find the specific organism of the disease in the cords of obviously syphilitic infants. When present they are to be found in the muscular coatings of the umbilical vein in sections taken from portions of the cord nearest to the umbilicus. In a large number of cases, however, in which, by examination of the placenta and post-mortem search in the liver, the organisms were found in those organs, prolonged and careful examination of numerous slides failed to show their presence in the cord.

### Tetanus Antitoxin as a Prophylactic.

For one reason or another the beneficent action of anti-tetanic serum is but rarely capable of proof, and much has been written of its failure to prevent disaster in many cases in which it has been used. It is therefore with satisfaction that we are able to record an observation of G. Lenthal Cheatle communicated to the *British Medical Journal*, in which he describes the case of a boy admitted to King's College Hospital after receiving a wound in the thigh from a street accident. This was purified and antitoxin administered. Fourteen days later tetanus bacilli in large numbers appeared in the discharge from the wound. The boy, however, exhibited no symptoms of the disease, the antitoxin having effectually protected him. The author thinks that this safe prophylactic measure should be used more generally than is now the case on patients who have met with accidents in which dirt from field, garden, or road has come into contact with the wound.

### Lutein Extract for Menstrual Deficiency.

According to the *Journal of the American Medical Association*, Dr. Ellice McDonald has for the past five years been making clinical observations on the effects of the administration of desiccated extract of the corpora lutea of cows in cases of scanty menstruation or of premature menopause. Ten of his twenty cases treated in this way were afflicted with disorders of natural origin, of whom seven were improved. The three unsuccessful ones were of long standing, with marked disturbance of the nervous system. Of the ten cases in which the menopause had been brought on by surgical means, one was made worse, four were somewhat improved, and five experienced no change in their condition. It would therefore seem that no relief can be obtained from lutein extract in surgical cases. It may, however, be of value in cases where the uterus and ovaries, or the uterus alone, are still present, but its chief results are to be obtained where there is scanty menstruation or premature menopause. In addition to oral administration of the extract in this latter class of patients, the author advises dilatation of the cervix with the use of the stem pessary after operation. He believes that the extract is useful after operations in pregnant women to minimise the risk of abortion; and this is still further indicated during the early weeks of pregnancy, since it is at this time that the corpus luteum has a definite effect. He is convinced that the corpus luteum has a distinct relation to the production of the menses, even if it is not the direct cause of this phenomenon.

### Tetanus from a Leech Bite.

Dr. Jose Carreras relates in the *Gaceta Medisa Catalana* that he was called on March 17 of this year to see a young man of good physique who showed all the signs of an uncomplicated pneumonia. He ordered an application of leeches *loco dolenti*, and the disease rapidly cleared up. In a few days, however, trismus appeared, and the patient was found to be suffering from tetanus, from which he died in the course of a week, despite energetic treatment with large doses of anti-tetanic serum supplied by the Institut Pasteur of Paris. This case of tetanus cannot, in the author's opinion, be accounted for in any other way than by supposing it to have resulted from the leeches' bites. The local chemist was not at fault, since he had no leeches in stock. Those used were obtained directly from a pond in the district. It is probable that at least one of them was infected with tetanus bacilli, with which the patient was inoculated at the time of biting. The author, therefore, advises that leeches should never be used which have not been kept a certain time in pure water.

